# Characterising the genomic landscape of two clinical risk groups in CD30+ T-cell lymphoproliferative disorders with cutaneous involvement: a multi-institutional retrospective study

**DOI:** 10.1016/j.ebiom.2026.106532

**Published:** 2026-10-27

**Authors:** Leonardo P.A. Biral, Veronica Russell, Maria Angelica Selim, Caroline Roth, Cassandra Love, Jennifer Shingleton, Dennis Owusu, Ayush Batra, Emily F. Mason, Alexandra Kovach, Catalina Amador, Jean L. Koff, David Jaye, Chee Leong Cheng, Andrew Evans, Raju Pillai, Sirpa Leppä, Eric D. Hsi, Sarah Ondrejka, Matthew McKinney, Chad M. McCall, Shelby Bettoney, Payal Sojitra, Andrew Evens, Amir Behdad, Carlos Galvez, Yuri Fedoriw, Elizabeth Thacker, Devang Thakkar, Clay Parker, Lanie Happ, Magdalena Czader, Rohit Gulati, Kikkeri N. Naresh, Sandeep S. Dave, Jie Wang

**Affiliations:** a Duke University, Durham, NC, United States; b Division of Hematopathology, Vanderbilt University, Nashville, TN, United States; c University of Nebraska, Omaha, NE, United States; d Department of Hematology and Medical Oncology, Winship Cancer Institute, Emory University School of Medicine, Atlanta, GA, United States; e Department of Pathology and Laboratory Medicine, Emory University, Atlanta, GA, United States; f Department of Anatomical Pathology, Singapore General Hospital, Singapore; g Department of Pathology and Laboratory Medicine, University of Rochester, Rochester, NY, United States; h Clinical Molecular Diagnostic Laboratory, City of Hope, Duarte, CA, United States; i University of Helsinki, Helsinki, Finland; j Helsinki University Hospital Comprehensive Cancer Center, Helsinki, Finland; k Mayo Clinic, Rochester, MN, United States; l Pathology and Laboratory Medicine Institute at Cleveland Clinic, Cleveland, OH, United States; m Robert Wood Johnson Medical School, Rutgers University, New Brunswick, NJ, United States; n Rutgers Cancer Institute, New Brunswick, NJ, United States; o Cleveland Clinic Florida, Weston, FL, United States; p University of Illinois, Chicago, IL, United States; q University of North Carolina, Chapel Hill, NC, United States; r Data Driven Bioscience, Durham, NC, United States; s Department of Pathology and Laboratory Medicine, Indiana University, Indianapolis, IN, United States; t Pathology Program, Translational Science & Therapeutics Division, Fred Hutchinson Cancer Center, Seattle, WA, United States

**Keywords:** Cutaneous T cell lymphoma (CTCL), Genomics, Mutational signatures, Gene expression profiles, Microenvironment

## Abstract

**Background:**

CD30+ T cell lymphoproliferative disorders (CD30+ T LPD) with cutaneous involvement comprise a diverse group of neoplasms with variable clinical outcomes. The molecular mechanisms underlying the prognostic differences in these diseases are poorly understood.

**Methods:**

In this multi-institutional retrospective study, 79 patients with CD30+ T LPD were evaluated. DNA and RNA sequencing were performed on skin biopsy samples to characterise molecular features. Patients were assigned to low- and high-risk groups based on treatment recommendations in clinical records. Pathogenic pathways, immune microenvironments, transcriptomic profiles, driver mutations, and mutation signatures were compared between the risk groups.

**Findings:**

Across risk groups, there was high mutational recurrence in genes involved in epigenetic regulation and intracellular signalling pathways. Mutational signature analysis revealed a shared prevalence of 5-methylcytosine deamination, UV exposure, defective mismatch repair, and POLE-associated proofreading deficiency. In contrast, gene set enrichment analysis showed that the high-risk group was enriched in pathways governing cell cycle progression, DNA damage response, and immune activation compared to the low-risk group. Furthermore, immune microenvironment deconvolution showed a higher abundance of resting dendritic cells in the low-risk group. Finally, high-risk patients exhibited significant overexpression of clinically relevant proteins, highlighting potential alternative treatment approaches for this population.

**Interpretation:**

Mutation analyses suggest diverse pathogenesis in these diseases. The high-risk and low-risk groups diverge in gene expression profiles and immune microenvironment, suggesting that these biological differences hold prognostic significance. Molecularly informed therapeutic strategies should be investigated prospectively through clinical trials.

**Funding:**

This work was supported by developmental funds of the 10.13039/100014228Duke Cancer Institute as part of the P30 Cancer Center Support Grant. Data generation was supported in an unrestricted fashion by the 10.13039/100000002NIH, Data Driven Bioscience, 10.13039/100016443Amazon and 10.13039/100006785Google. SSD was supported by the Wellcome Trust Foundation and the 10.13039/100000002NIH.

**Research in context**

**Evidence before this study**

CD30+ T-cell lymphoproliferative disorders (LPD) with cutaneous involvement represent a diverse group of malignancies sharing overlapping histopathologic features but exhibiting highly variable clinical outcomes. Notably, histologic evaluation alone is insufficient to predict disease aggressiveness. We searched PubMed, Google Scholar, and the AI-assisted medical platform OpenEvidence for peer-reviewed articles published from database inception to January 2026. Search terms included combinations of “CD30”, “cutaneous T-cell lymphoma”, “anaplastic large cell lymphoma”, “lymphomatoid papulosis”, and “genomics”. OpenEvidence was leveraged to identify specialised, smaller series analysing CD30 positivity, genomic alterations, and clinical outcomes. The compiled evidence confirmed marked clinical heterogeneity despite overlapping histopathologic findings. Furthermore, the search revealed a dearth of genomic studies on cutaneous CD30+ T-cell LPD where the precise integrated clinicopathologic diagnosis may be uncertain, and that include clinical correlation, treatment patterns and survival outcomes.

**Added value of this study**

This is an in-depth genomic study of CD30+ T LPD with cutaneous involvement. Rather than subdividing this disease spectrum according to idealised diagnostic categories, we address the more clinically relevant question of what molecular differences distinguish aggressive from indolent cases. Because more intensive therapies are typically reserved for more aggressive disease, we use treatment recommendations as a surrogate for clinical aggressiveness, stratifying the cohort into high- and low-risk groups. Survival analysis confirms significantly poorer overall survival in the high-risk group. Although mutational features are shared across risk groups, high-risk cases exhibit significantly increased expression of genes involved in cell cycle progression, DNA damage response, and immune activation. In addition, immune cell deconvolution of bulk transcriptomic data reveals significantly higher proportions of resting dendritic cells among patients within the low-risk group, consistent with a less inflammatory tumour microenvironment.

**Implications of all the available evidence**

This multicentre retrospective genomic study of CD30+ T LPD identifies significant differences in gene expression profiles and the immune microenvironment between low- and high-risk cases with cutaneous involvement. These findings demonstrate that distinct molecular features correlate with the clinically observed differences in disease behaviour. The study provides clinically relevant insights and helps inform the development of more rational, targeted therapeutic strategies, particularly for patients with high-risk disease, who continue to represent an area of unmet clinical need.

## Introduction

CD30+ T cell lymphoproliferative disorders with cutaneous involvement (CD30+ T LPDs) comprise a heterogeneous group of lymphoid malignancies with overlapping histopathologic features but highly variable clinical behaviour. These encompass CD30+ mycosis fungoides (MF) (including cases with large cell transformation), nodal anaplastic large cell lymphoma (nALCL) with secondary skin involvement, and the primary cutaneous CD30+ LPDs that include lymphomatoid papulosis (LyP) and primary cutaneous anaplastic large cell lymphoma (cALCL). Clinical presentation ranges from indolent disease, often managed with observation or skin-directed therapy to aggressive disease requiring systemic therapy or radiotherapy.

While LyP is indolent, and nALCL is aggressive, the remaining entities exhibit substantial clinical variability, underscoring the need to define biological drivers of disease heterogeneity. Although CD30+ MF has been associated with worse outcomes compared to CD30-negative disease,^1^ its clinical course remains highly variable, with some cases requiring only skin-directed therapy and others requiring aggressive systemic therapy either early in the disease course or later with histologic transformation.^2^ Diagnostic overlap is also common, with cases frequently classified as CD30+ T LPD not otherwise specified (NOS) and treatment decisions are guided by clinical severity rather than precise subtype. While CD30 is a unifying histopathologic feature and a therapeutic target of brentuximab vedotin, it does not consistently predict treatment response and is an unreliable prognostic marker.^3^ CD30 clearly has significant limitations as a biomarker in these disorders.

In clinical practice, patients may be considered low- or high-risk partly based on disease stage or burden, which includes the extent of skin involvement, tumours, lymphadenopathy, or other extracutaneous disease. Disease-related wounds and systemic symptoms are also features of aggressiveness, but are not captured by cutaneous lymphoma staging systems. Collectively, these features determine therapeutic intensity, but prior studies examining the molecular landscape of CD30+ T LPDs have not incorporated clinical risk stratification.

To address this gap, we included 79 patients with CD30+ T LPD with cutaneous involvement and characterised their clinical history and molecular features. Specifically, we examined differences in survival, mutations in driver genes, mutational signatures, gene expression, pathways, and tumour immune microenvironment between patients requiring aggressive systemic therapy (high-risk) and those managed without it (low-risk). Reflecting real-world diagnostic practice where a skin biopsy showing a CD30+ T LPD requires clinical correlation for definitive classification, our cohort includes CD30+ MF, transformed MF, nALCL with secondary skin involvement, and primary cutaneous CD30+ LPDs that include LyP and cALCL. Because treatment decisions are ultimately driven by clinical behaviour rather than formal diagnostic category alone, these entities were analysed collectively to identify shared and divergent molecular features across risk groups.

## Methods

### Inclusion criteria, sample collection, library preparation and sequencing

Only cases of cutaneous involvement by a CD30+ T-cell lymphoproliferative disorder were included in this study. Eligible cases required the availability of archival biopsy material in excess of clinical diagnostic needs; however, a subset of these cases did not yield sufficient quality or quantity of DNA/RNA for subsequent genomic analysis. Cases with no available clinical information were excluded.

Study samples were obtained from The Atlas of Blood Cancer Genomes (ABCG) project, an international collaborative effort that aims to genomically profile all blood cancers (manuscript in preparation). The study cohort included diagnostic formalin-fixed paraffin-embedded (FFPE) tumour biopsies collected from 79 patients with CD30+ T LPD at 12 academic institutions (Supplementary Figure S1A). All diagnostic tissue biopsies were reviewed and diagnoses confirmed by expert, panel-based pathology review as part of the ABCG consortium. All cases were CD30 positive as confirmed by immunohistochemistry. All nucleic acid extractions and sequencing library preparations were performed with Duoseq (Research Kit EPXv3, Data Driven Bioscience, Durham, NC) following the manufacturer's protocol. All libraries were sequenced on the Illumina platform and analysed according to manufacturer recommendations.

Whole transcriptome RNA sequencing (RNAseq) was successfully performed in a total of 44 patients (Supplementary Figure S1B). Whole-exome DNA sequencing (WES) was successfully performed in 37 patients to a mean depth of 31×. Additionally, targeted panel DNA sequencing^4^ was performed for driver genes in 35 patients to a mean depth of 61×. The union of the 37 patients that underwent WES and the 35 patients that underwent targeted sequencing was 38 patients (Supplementary Figure S1C, Supplementary Table S1). The mean combined DNA sequencing coverage for driver genes was estimated at 95×.

### Clinical and pathology review

Available clinical charts were reviewed for patient demographics, clinical and pathologic diagnosis, CD30 positivity, and treatment history. If patients were recommended treatment at any time with high-risk therapies (e.g. cytotoxic chemotherapy, romidepsin, pralatrexate, alemtuzumab, or bone marrow transplant), they were assigned to the high-risk group. If patients received topical therapies, radiation, light therapy, or other systemic therapy not included above, they were assigned to the low-risk group. Risk stratification was based on treatment recommendations as the use of more toxic systemic therapies generally reflects greater disease aggressiveness. Clinical staging was not used due to high-risk clinical features not being captured by staging systems, the heterogeneity of staging systems across diagnoses, some cases being unstageable (e.g. CD30+ LPD NOS, and LyP), and the evolving nature of stage in cutaneous T-cell lymphomas (CTCLs).^5^ To incorporate objective stage-related measures, we additionally assessed the presence of tumours and diffuse skin involvement in cases with available clinical descriptions.

### DNA sequencing analysis

FASTQ files^6^ containing DNA sequencing reads were trimmed using Trimmomatic^7^ (v0.39) in paired end mode to remove adaptor sequences and low-quality reads. Using Sentieon BWAmem^8^ (v201911) with the default settings, DNA reads were aligned to the human genome (GRCh38.p12, with a PAR mask on chrY). PCR duplicate reads were marked using Picard (v2.8.1) (http://broadinstitute.github.io/picard). Picard, FASTQC (v0.11.8) (www.bioinformatics.babraham.ac.uk/projects/fastqc), and SAMtools^9^ (v1.13) were used to extract quality control metrics.

### Variant calling analysis

Variant calling analysis was performed using DNA reads from exome samples and targeted panel sequencing where available. Variants were called using a pipeline that uses the union of variants called by each of Strelka2^10^ (v2.9.10), DeepVariant^11^ (v1.1.0), and Sentieon Haplotyper^8^ (v201911). Variants with a population frequency of greater than 0.01 reported in the gnomAD^12^ databases were excluded. The remaining variants were reviewed using Integrative Genomics Viewer (IGV).^13^ The oncoprint was generated using genes that were classified as cancer-associated according to public databases, including OncoKB and COSMIC. Mutational signatures were assigned using the COSMIC^14^ SigProfilerAssignment^14^ (RRID: SCR_026899).

### RNA sequencing analysis

FASTQ files containing RNA-Seq reads were trimmed using Trimmomatic^7^ (v0.39) in paired end mode to remove Illumina specific adaptor sequences and low-quality reads. STAR aligner^15^ (v2.7.1) was utilised for mapping RNA reads to the human genome and transcriptome (GRCh38.p12, with a PAR mask on chrY). Reads that had at least one primary alignment to the human genome were extracted using SAMtools^9^ (v1.13) and additionally filtered based on manufacturer recommendations using GATK^16^ (v4.1.2.0). Transcript quantification was performed using salmon^17^ (v1.2.1), and the transcript-level data were summarised to the gene level using the tximport^18^ (v1.14.2) library in R. The result was a matrix representing raw read counts per gene per patient sample. This matrix was restricted to include only the expression of the protein-coding genes captured by exome sequencing. Genes with missing or uniformly low expression were excluded from further analysis and the remaining expressed genes were normalised across the entire sample set in R using the DESeq2^19^ (v1.40.2) tool. Differential expression analysis was also performed with the DESeq2 (v1.40.2) tool and the differential expression heatmap was generated with the ComplexHeatmap R package^20^ using genes that had a nominal p-value of less than 0.01 and a Benjamini-Hochberg adjusted p-value of less than 0.1. Gene Set Enrichment Analysis^21^ (GSEA) was performed using the Molecular Signature Database^22^ (MSigDB). Statistical significance in GSEA comparisons was calculated with permutation tests and the Benjamini-Hochberg multiple testing correction was retroactively applied. All GSEA p-values reported are corrected for multiple testing. Tumour microenvironment cell fraction estimation was performed using FARDEEP.^23^

**Fig. 1:**
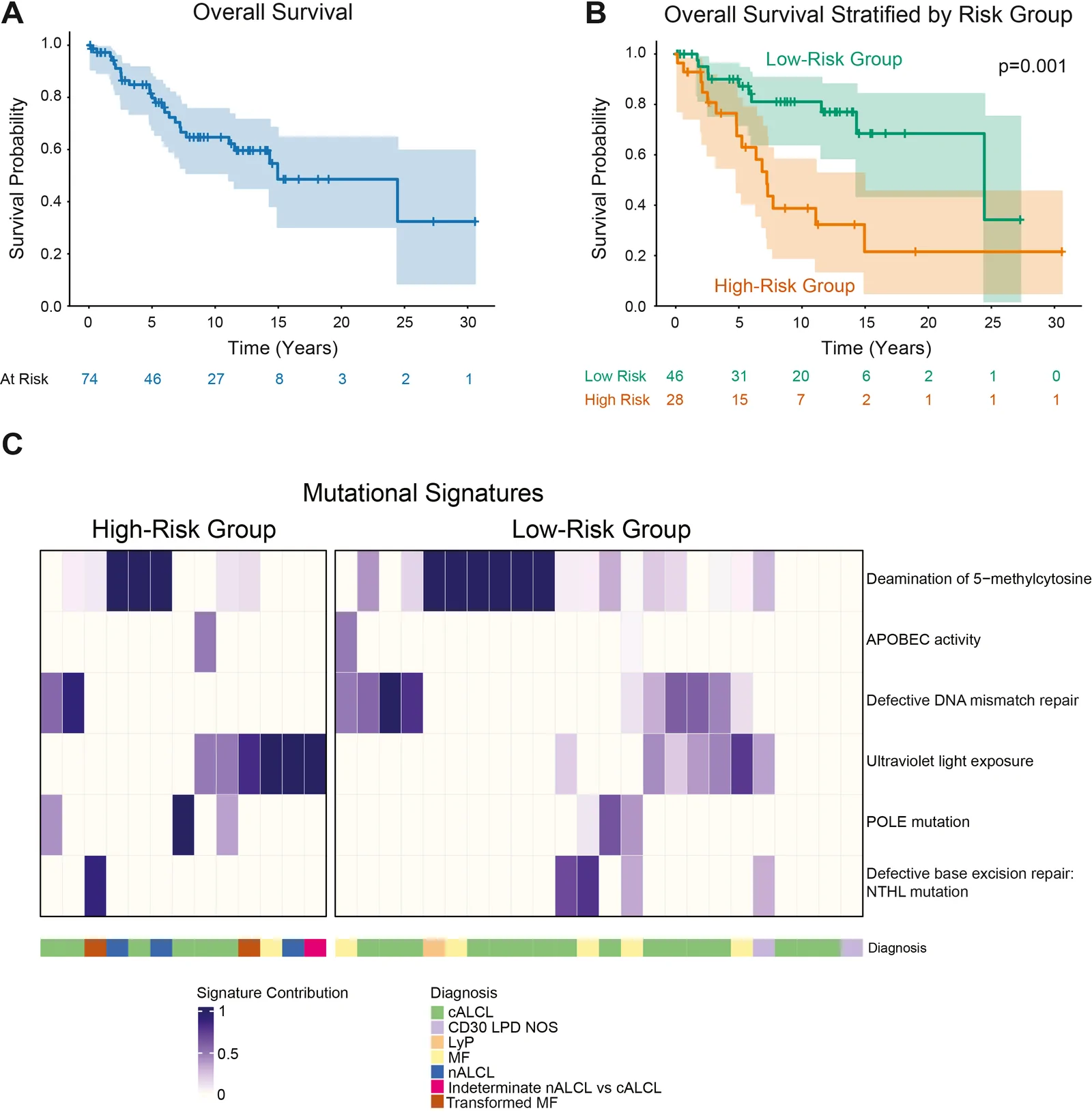
**Survival and mutational landscape in CD30+ CTCL. (A)** Overall survival Kaplan–Meier curve of the 74 patients in the cohort with survival outcome data. Number of patients at risk and 95% confidence intervals (CI) shown. **(B)** Overall survival Kaplan–Meier curves stratified by clinical risk group of the 74 patients in the cohort with survival outcome data. Number of patients at risk and 95% CIs shown. We observe significant differences in survival between the low (green, n = 46) and high (orange, n = 28) risk groups (p = 0.001 [log-rank]). **(C)** Heatmap of selected mutational signatures with the most hits in CD30+ CTCL. Heatmap indicates the relative contributions of various mutational signatures (right) to each sample. Samples are grouped by the clinical risk group (high-risk n = 13, low-risk n = 24) and annotated by diagnosis (legend below). cALCL, cutaneous anaplastic large cell lymphoma. CD30 LPD NOS, CD30+ lymphoproliferative disorder (not otherwise specified). LyP, lymphomatoid papulosis; MF, mycosis fungoides; nALCL, nodal anaplastic large cell lymphoma.

**Fig. 2:**
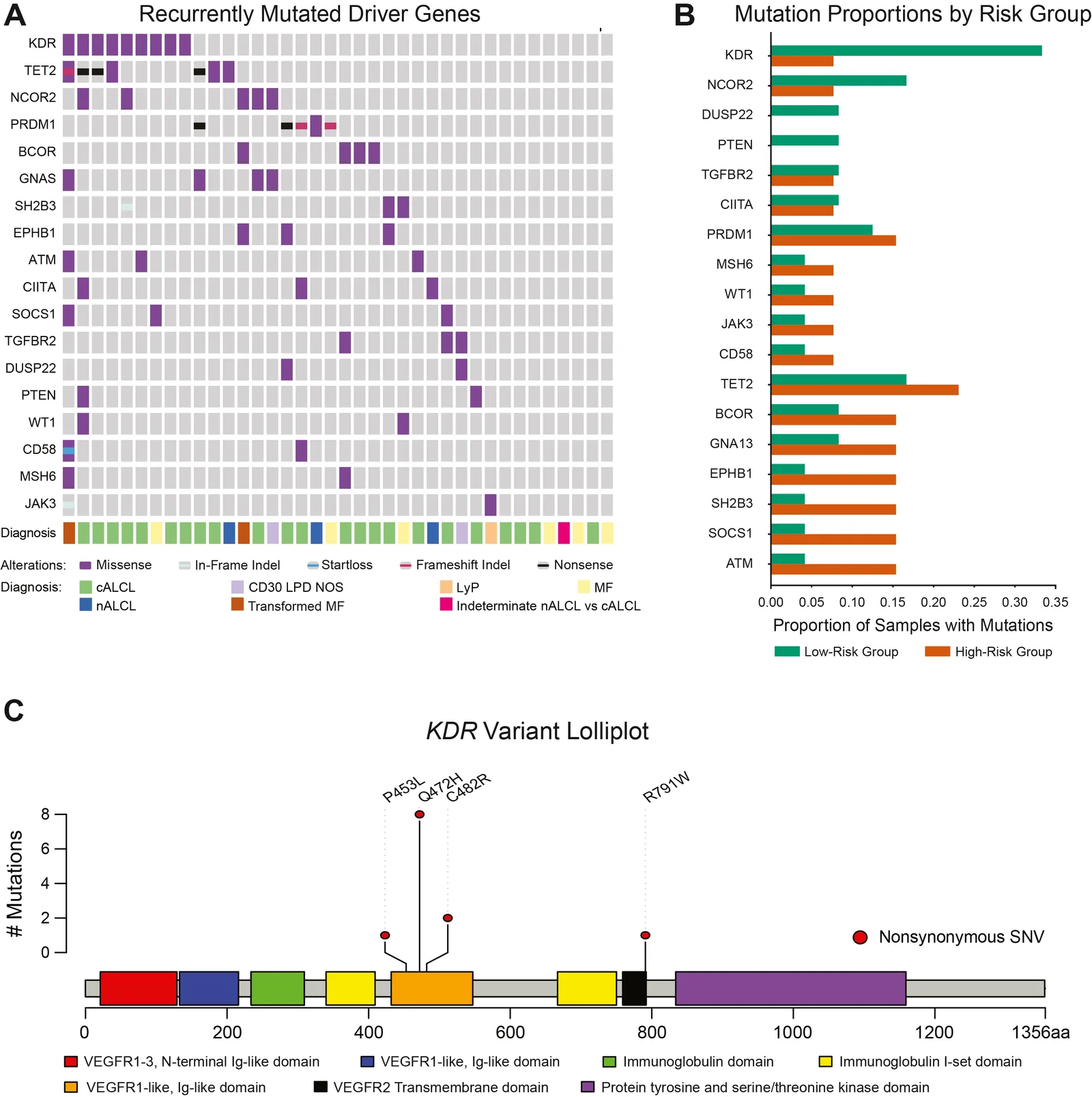
**Clinical risk group survival and mutational comparisons. (A)** Oncoprint of the most recurrently mutated, putative driver genes in CD30+ CTCL, colour-coded by type of alteration. Below the oncoprint, samples (n = 38) are annotated by diagnosis. **(B)** Grouped barchart showing differences in mutational proportions between samples of the two clinical risk groups. **(C)** Lolliplots of variants found in *KDR*. The number of times each variant appeared in the cohort is indicated by the height of the stick. Functional domains are indicated by coloured boxes and described below the lolliplot. cALCL, cutaneous anaplastic large cell lymphoma. CD30 LPD NOS, CD30+ lymphoproliferative disorder (not otherwise specified). LyP, lymphomatoid papulosis; MF, mycosis fungoides; nALCL, nodal anaplastic large cell lymphoma.

**Fig. 3:**
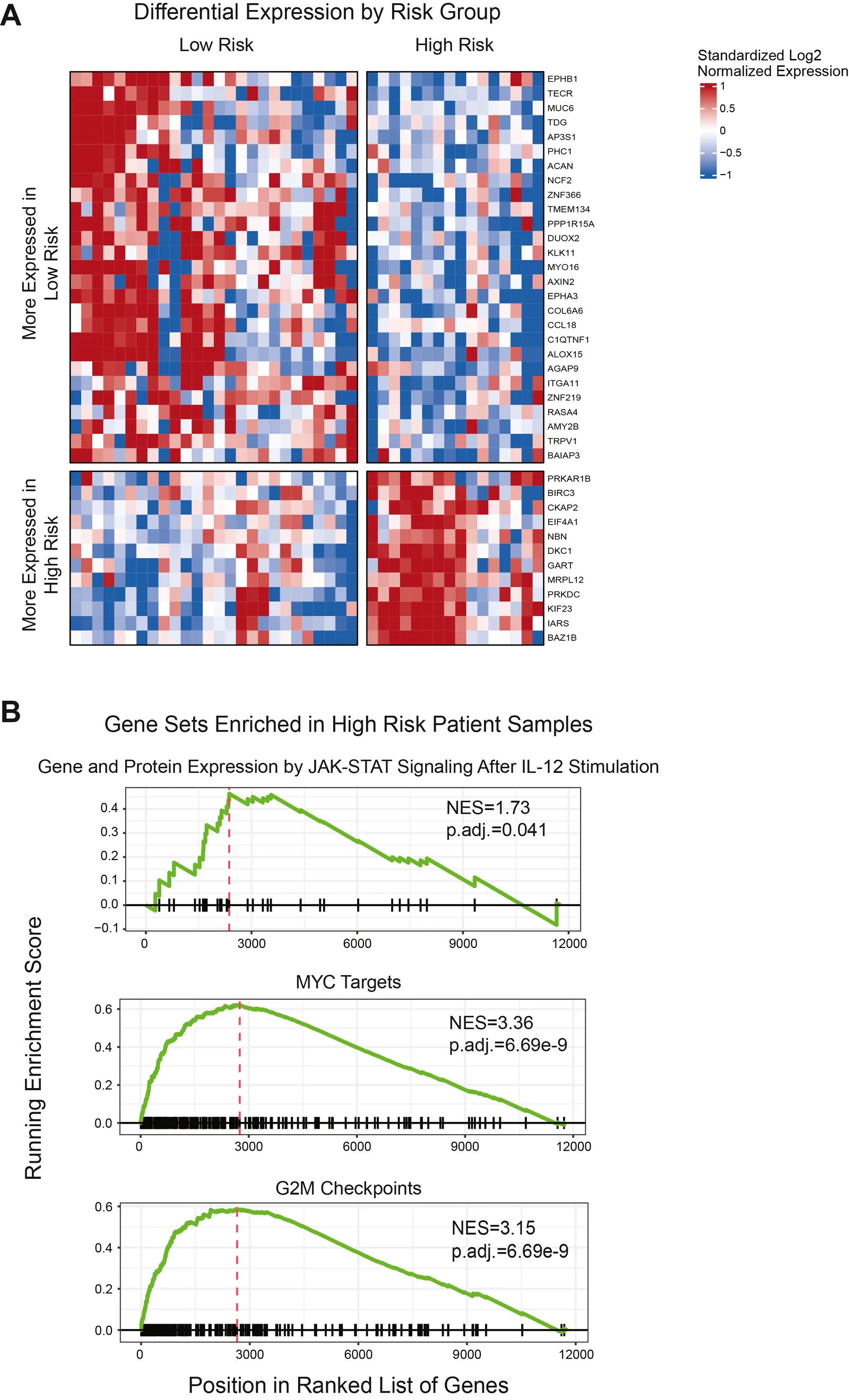
**Clinical risk group expression comparisons. (A)** Heatmap showing standardised log2-normalised expression values of genes (right) that had significant differential expression between the two clinical risk groups (p-adj.<0.1). Heatmap is column-split by clinical risk group and row-split by the directionality of the gene expression values. **(B)** GSEA enrichment plots showing significant enrichment of gene sets involved in JAK/STAT signalling (NES = 1.73, p-adjusted = 0.041), MYC targets (NES = 3.36, p-adj. = 6.69e-9, and G2M checkpoint genes (NES = 3.15, p-adjusted = 6.69e-9) in the high clinical risk group. NES, normalised enrichment score.

### Fusion analysis

Fusions were called using Arriba^24^ (v2.1.0). To retain high-confidence fusion events for downstream analysis, we applied a multi-step filtering strategy. First, we selected fusions annotated in the Mitelman database,^25^ or those that were called by Arriba with high confidence. We then excluded fusion events lacking annotated transcript IDs for either fusion partner. To ensure we selected only biologically and technically supported events, we calculated a total support metric for each fusion as the sum of split reads from both partners and discordant mate-pair support. Fusions with a total support count below five reads were excluded from further analysis. This threshold was chosen to remove likely false-positive calls arising from mapping artifacts, low-level transcriptional noise or library-specific technical artifacts.

### Statistics

Due to the highly specific histopathological and immunophenotypic criteria required for inclusion, a formal sample size determination was not performed. The cohort size was determined by the available archival tissue meeting the specific diagnosis of a T-cell lymphoproliferative disorder with documented CD30 expression and availability of overall survival data. Kaplan–Meier curves were generated using the lifelines Python library.^26^ The statistical significance of Kaplan–Meier curve stratification was computed with a two-tailed log-rank test. The statistical significance of the difference in mutation rates of each oncoprint gene between patients in the high- and low-risk groups was calculated with a Fisher's exact test. Statistical significance of cell type abundance in high-vs. low-risk groups was calculated with a Mann–Whitney U-test. To identify pathway involvement of mutated genes, we used Enrichr^27^ on the default settings.

### Ethics

Protocol approval for this retrospective study, including a waiver of individual patient consent and HIPAA authorisation, was obtained from the Duke Institutional Review Board for the local chart review and sample analysis (Pro00016490). Archival tissue samples and clinical data from the collaborating centres were provided in a completely de-identified format, adhering to established material transfer and data-sharing agreements, and is not considered human subjects research.

### Role of funders

Data generation was supported in an unrestricted fashion by the NIH, Data Driven Bioscience, Google and Amazon. The funders had no role in study design, data collection, data analyses, interpretation or writing of the report.

## Results

We included a total of 79 patients with archival skin biopsies showing CD30+ T cell LPD for this study. Their clinical characteristics are described in Table 1 and Supplementary Table S2. 57% of patients were male, with median age of diagnosis of 62 years. Of the 60 cases with available information about race, 48 (80%) were white, seven (12%) were black, and five were Asian (8.3%). There were 39 patients with cALCL, five with LyP, 17 with MF, six with transformed MF, seven with nodal ALCL, four with CD30+ LPD NOS, and one with ALCL that could not be definitively classified as primary cutaneous ALCL with nodal involvement or nALCL with secondary cutaneous involvement. Of the LyP cases, four were type A and one was type C. Among the nALCL cases, two were ALK-positive and five were ALK-negative.

**Table 1:** Clinical characteristics of CD30+ CTCL patient cohort.

Characteristic	Number of cases with available data	Value^a^
Age at diagnosis	75	62 (52, 70)
Patient sex	63	
Female		27 (43%)
Male		36 (57%)
Race	60	
Asian/Pacific Islander		5 (8.3%)
Black		7 (12%)
White		48 (80%)
B symptoms present at diagnosis	16	1 (6.3%)
Diagnosis	79	
cALCL		39 (49%)
CD30+ LPD NOS		4 (5.1%)
LyP		5 (6.3%)
MF		17 (22%)
nALCL		7 (8.9%)
Indeterminate (nALCL vs. cALCL)		1 (1.3%)
Transformed MF		6 (7.6%)
Risk group	79	
High Risk		28 (35%)
Low Risk		49 (62%)
Missing		2 (2.5%)

LyP, lymphomatoid papulosis; MF, mycosis fungoides; nALCL, nodal anaplastic large cell lymphoma.

cALCL, primary cutaneous anaplastic large cell lymphoma. CD30+ LPD NOS, CD30+ lymphoproliferative disorder (not otherwise specified).

a Median (Q1, Q3); n (%).

For the 74 patients with available treatment history and survival data, the median follow-up time was 11.7 years (95% CI: 8.66–13.6, Fig. 1A). Among these 74 patients, 46 met the criteria for the low-risk group, and the remaining 28 were in the high-risk group. The median overall survival was significantly greater for those in the low-risk group, with median overall survival 24.4 years compared to 7.21 years in the high-risk group (Fig. 1B, p = 0.001 [log-rank]). While all patients with LyP were in the low-risk group, and patients with nALCL were in the high-risk group, patients with cALCL, MF, and CD30+ T LPD NOS were represented in both risk groups. The statistically significant difference in survival between the two groups was preserved when LyP and nALCL cases were excluded (p = 0.001 [log-rank]), confirming it was not these diagnoses alone that drove the survival difference (Supplementary Figure S2A). Using tumours and diffuse skin involvement as stage-based high-risk features, concordance with treatment-based stratification was 67% (Supplementary Figure S2B). Skin staging tended to re-classify low-risk patients as high-risk due to tumour-stage lesions, and unlike treatment-based stratification, did not distinguish patient groups with significantly different overall survival outcomes (p > 0.05 [log-rank test]) (Supplementary Figure S2C).

### Mutational signatures in CD30+ T cell lymphoproliferative disorders

We next sought to characterise genetic drivers of the disease and identify biological differences between the groups. To identify gene mutation signatures and frequently mutated genes, we used the subset of 38 patients that passed DNAseq quality control filtering (Supplementary Figure S1).

We began examining the mutational profiles in these tumours by comparing the identified genetic events to previously described mutation signatures.^14^, ^28^ Both clinical risk groups were analysed for the gene mutation signatures (Fig. 1C, Supplementary Table S3). The UV light gene signature, which refers to C > T transitions at dipyrimidine sites reflective of DNA damage due to UV radiation, is of particular interest in this study of cutaneous pathology. Of all variants in the cohort, 11.9% were associated with the UV mutational signature. Out of 38 cases, 13 (34%) had a UV mutational signature and there were no significant differences in this mutational pattern between the two risk groups (p = 0.47 [Fisher's exact]). Of the three nALCL cases included in this analysis, one was positive for UV mutational signature. Of the 23 cases of cALCL, seven (30.4%) had a UV mutational signature. Finally, the mutational profile indeterminate case of nodal versus cutaneous ALCL also had a high percent identity with the UV mutational signature.

Gene signature analysis showed frequent involvement of deamination of 5-methylcytosine with a signature contribution in 24 (63%) cases. Deamination of 5-methylcytosine causes C > T transition mutations at CpG dinucleotides when the resulting T:G mismatch is not corrected efficiently through mechanisms such as DNA mismatch repair and base excision repair.^29^, ^30^ Eight (33%) of these cases also had defective DNA mismatch repair mutational patterns, and five (21%) had defective base excision repair.

*POLE* mutation-associated gene signatures also occurred in a subset of cases. This gene signature is associated with pathogenic variants in the exonuclease domain of the DNA polymerase epsilon (*POLE*) gene, which can result in defective DNA replication proofreading and may be predictive of the utility of immune checkpoint inhibitors.^31^ No mutation signature was statistically significantly more represented in one clinical risk group compared to the other indicating shared mutational profiles across all patients in the cohort.

### Genetic drivers of CD30+ T cell lymphoproliferative disorders

We further examined the most frequently altered genes using DNA sequencing. Using approaches similar to those previously described,^32^, ^33^ we found recurrent variants in 18 likely driver genes (Fig. 2A, Supplementary Table S4). Of the 38 cases with available DNA sequencing, 30 cases (79%) had at least one variant in these 18 genes. Three cases did not have any variant found by WES, which could reflect the presence of non-exonic drivers or insufficient sensitivity of our approach. No statistically significant differences in mutation frequency were found in any gene when comparing the two clinical risk groups (p > 0.05 [Fisher's exact]; Fig. 2B).

*KDR* and *TET2* emerged as the most frequently altered genes, occurring in 24% and 18% of cases, respectively. *KDR*, also known as *VEGFR2*, is a receptor for VEGF, a signalling protein that promotes angiogenesis. In our study, 12 *KDR* variants were identified (Fig. 2C). Eight (67%) of them were p.Gln472His, a single nucleotide variant and functional polymorphism that is a known germline variant included in OncoKB.^34^ There was a trend toward *KDR* mutations being more prevalent in the low-risk group compared to the high-risk group, but this difference was not statistically significant (p > 0.05 [Fisher's exact]) (Fig. 2B).

The next most frequently altered gene was *TET2*, an epigenetic modifier that catalyses the process of DNA demethylation, regulating DNA expression (Figs. 1C and 2B). *TET2* is among several genes involved in epigenetic regulation identified in our dataset. These genes (*TET2*, *NCOR2*, *PRDM1,* and *BCOR*) were frequently mutated, with mutations found in 18/38 cases (47%) (Supplementary Figure S3). Most common were mutations in tyrosine kinases or signalling genes that mediate cellular proliferation through intracellular signalling pathways (*KDR*, *GNAS*, *EPHB1*, *TGFBR2*, and *JAK3*). These mutations occurred in 19 cases (50%) (Supplementary Figure S4). Gene alterations in suppressors of cell signalling (*SH2B3*, *SOCS1*) occurred in six cases (16%) (Supplementary Figure S5), and mutations in additional tumour suppressors (*PTEN*, *DUSP22*, and *WT1*) affected five cases (13%) (Supplementary Figure S6). DNA damage response genes (*ATM*, *MSH6*) were affected in four cases (11%) (Supplementary Figure S7), and mutations in genes allowing immune evasion (*CIITA*, *CD58*) also occurred in four cases (11%) (Supplementary Figure S8).

Mutations spanned a range of alteration classes, including missense variants, frameshift and in-frame indels, nonsense mutations, and start-loss events. Notably, truncating mutations (i.e., mutations that result in loss of function) were frequent in *TET2* and *PRDM1*, genes with known tumour suppressor activity.

### Gene fusion analysis

Beyond mutations in driver genes, we also evaluated the cases for oncogenic fusions. There were 44 cases available for gene fusion analysis, and we found four cases with in-frame fusion transcripts. *NPM1*::*ALK* was identified in a case of nALCL, *NPM1*::*TYK2* and *PARG*::*BMS1* were each identified in cases of cALCL, and *PRMT1*::*TYK2* was identified in a case of transformed MF. All cases were in the high-risk group.

### Gene expression and mutation over-representation analyses

To better understand gene expression differences associated with clinical outcome, we compared gene expression profiles between patients in the low- and high-risk groups. The full list of differentially-expressed genes (p < 0.01) is found in Supplementary Table S5. Fig. 3A illustrates a heatmap of the top differentially expressed genes between the two clinical risk groups. A distinct transcriptional signature emerged in high-risk patients, characterised by elevated expression of genes associated with cell cycle progression, DNA damage response, and immune activation. These included *CKAP2, SSBP1, PDE4D, BIRC3, IL2RA, B2M, NBN, ERO1A, CKAP5, IARS, POLR2B, CUL1, PRKDC, KIF23, BAZ1B*, and *DGKG*. Many of these genes are involved in key oncogenic processes such as mitotic spindle assembly (*CKAP2, KIF23*), DNA repair (*NBN, PRKDC*), and antigen presentation (*B2M, IL2RA*), suggesting a hyperproliferative and immunologically active tumour phenotype in the high-risk group.

To characterise the biological processes associated with both high-risk disease and mutations in *KDR* (the most frequently altered gene in our cohort) we performed GSEA. In *KDR*-mutated cases, we observed significant enrichment of pathways related to G protein-coupled receptor activity (including amine receptor activity) (NES = −3.49; p-adj. = 6.32e-8), insulin-like growth factor binding (NES = −2.05; p-adj. = 0.0478), and cyclic nucleotide/Gαs signalling (NES = −2.24; p-adj. = 6.02e-3) (Supplementary Table S6). These findings support a functional impact of *KDR* mutations and are consistent with activation of intracellular signalling pathways. Notably, the enrichment of *KDR* mutations in the low-risk group suggests that additional mechanisms underlie the high-risk phenotype.

Consistent with prior studies, intracellular signalling pathways such as JAK/STAT and PI3K/AKT are active in our cohort. However, mutations in canonical components of these pathways were relatively infrequent: we identified two cases with *JAK3* mutations and three with *SOCS1* mutations. Similarly, *PTEN*, a key negative regulator of PI3K/AKT signalling, was mutated in only two cases, with no additional recurrent mutations directly linked to PI3K/AKT activation. To assess pathway involvement of mutated genes, we performed an overrepresentation test using Enrichr.^27^ This analysis demonstrated significant enrichment of both the Hallmark JAK/STAT signalling (p = 5.685e-3) and the Reactome PI3K signalling (p = 9.69e-13) pathways. Exploring mutation pathway enrichment in the risk groups separately revealed significant enrichment of the Reactome PI3K pathway only among low-risk cases (p = 4.78e-13) and the Hallmark JAK/STAT pathway only among high-risk cases (p = 1.77e-3). Together, these results indicate that, despite few recurrent gene-level mutations, both pathways are functionally active and may differ between groups in our cohort.

Next, GSEA was used to assess pathway level differences between the risk groups (Fig. 3B, Supplementary Table S7). GSEA confirmed significant enrichment of several JAK/STAT target genes in the high-risk group compared to the low-risk group (NES = 1.73; p-adj = 0.041). This pathway is canonically activated by cytokine and interferon receptor engagement. High-risk cases also showed marked enrichment of proliferative and cell cycle–associated programs. Specifically, the Hallmark MYC Targets and Hallmark G2M Checkpoint pathways were significantly enriched (NES = 3.36, p-adj. = 6.69e-9; NES = 3.15, p-adj. = 6.69e-9, respectively) (Fig. 3B, Supplementary Table S7). These findings persisted even after excluding LyP and nALCL cases, indicating the observed differences are not driven by these subtypes. The MYC Targets gene set reflects transcriptional activation of genes regulated by the oncogenic MYC transcription factor, a key driver of proliferation and metabolic reprogramming, while enrichment of the G2M Checkpoint gene set indicates increased cell cycle progression in high-risk disease.

In contrast, low-risk cases were enriched for G protein-coupled receptor signalling, consistent with the earlier finding of enrichment of *KDR* mutated cases in the low-risk group (NES = −2.98; p-adj. = 6.69e-9) (Supplementary Table S7). The low-risk cases were also enriched for genes downregulated by KRAS signalling, consistent with a less proliferative phenotype (NES = −1.87; p-adj. = 1.33e-3) (Supplementary Table S7).

In addition to transcriptome-wide analyses, we performed a prespecified analysis of *AURKA*, a serine/threonine kinase regulating the G2/M transition, based on prior literature.^35^ As this hypothesis-driven, single-gene comparison was defined a priori, multiple testing correction was not applied. Although *AURKA* was not identified in the multiple-testing–adjusted GSEA discovery analysis, its expression was significantly higher in the high-risk group (fold change = 1.86; p = 0.0113 [Wald]) (Supplementary Table S5).

Together, these findings suggest that increased proliferative signalling, cytokine-driven signalling, and MYC-driven transcriptional programs contribute to the more aggressive clinical phenotype observed in the high-risk group. These transcriptomic differences underscore the biological divergence between high- and low-risk cases and support a model in which hyperproliferative and inflammatory signalling pathways drive adverse outcomes in CD30+ T LPD.

### Immune microenvironment cell deconvolution

To examine whether differences in the tumour microenvironment (TME) are associated with the divergent clinical outcomes observed between patient groups, we performed immune cell deconvolution using bulk transcriptomic data. Fig. 4A displays estimated proportions of 22 immune cell populations across patients, grouped by risk (see Supplementary Table S8 for full matrix). The inferred immune landscapes reveal immune cell compositional differences between high-risk and low-risk cases, with notable variation across several innate and adaptive immune compartments.

**Fig. 4:**
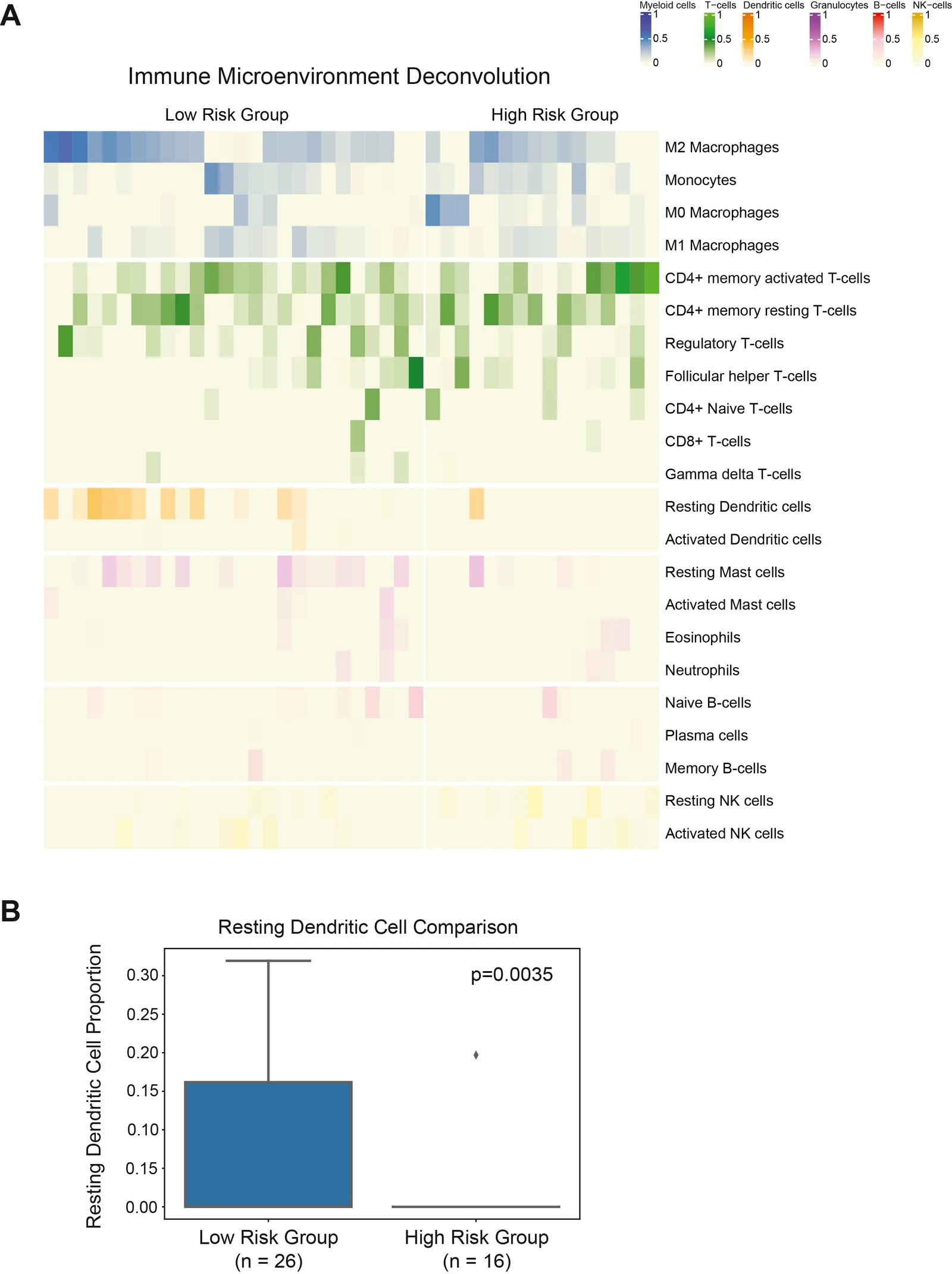
**The immune microenvironment of CD30+ CTCL. (A)** The heatmap shows the relative contribution of each immune cell type (right) to samples column-split by clinical risk group. **(B)** The comparative box plot visualises the proportions of resting dendritic cells by clinical risk group (low-risk n = 26, high-risk n = 16, p = 3.5e-3 [Mann–Whitney U]).

There was a significantly higher abundance of resting dendritic cells in patients in the low-risk clinical group compared to the high-risk group (p = 0.0035 [Mann–Whitney U]) (Fig. 4B). The difference in resting dendritic cells across risk groups was preserved when LyP and nALCL cases were excluded.

## Discussion

This study presents a comprehensive genomic analysis of 79 cases of CD30+ T LPD with cutaneous involvement. A significant limitation of prior studies is small sample size, and use of targeted gene sequencing panels. A prior study that included 86 cases of CD30+ LPD did not include any genomic analysis of the cases.^36^ This study, which includes genomic analysis and clinical correlation, shows the sheer diversity of pathogenic mutations, gene expression, mutational signatures, and tumour immune microenvironments that are associated with the biology in these diseases.

A central aim was to identify molecular features distinguishing clinically defined high- and low-risk cases of CD30+ T LPD. While mutational signatures and overall gene mutation profiles were similar between groups, we observed clear differences in gene expression. This discordance may be explained by the high prevalence of mutations in epigenetic regulators, identified in 47% of cases, which can drive transcriptional changes independent of underlying mutational differences.

Although epigenetic dysregulation is well recognised in T cell malignancies, it has been underexplored in CD30+ T LPDs, where prior studies have been limited in size and scope.^37^ In our cohort, alterations in epigenetic regulatory genes emerged as a prominent feature, with *TET2* representing the second most frequently mutated gene. While our study includes MF, which is well recognised for epigenetic dysregulation,^38^, ^39^ we also found variants in epigenetic regulatory genes across cases of primary cALCL, nALCL, and CD30+ T LPD NOS, highlighting a potentially underrecognised role for epigenetic mechanisms in this disease spectrum.

Among the recurrent epigenetic regulator variants identified in this study, *TET2* and *PRDM1* have well-established roles in T cell malignancies and primary cutaneous CD30+ T LPD, respectively.^39^, ^40^, ^41^, ^42^, ^43^, ^44^, ^45^, ^46^ In contrast, *BCOR* and *NCOR2* are less well characterised, but appear relevant in this context. *BCOR* is recurrently mutated in extranodal NK/T-cell lymphoma and other nodal lymphomas, particularly mature T-cell lymphomas, but has not been reported as recurrent in cutaneous T-cell lymphoma or CD30+ T LPD.^47^, ^48^ Although the pathogenic role of *NCOR2* in T-cell malignancies remains unclear, its mutations have been sporadically described in small series of CD30+ lymphoproliferative disorders,^49^ and an *NCOR2*::*ROS1* fusion has been identified in ALK-negative ALCL.^50^ Together, these findings suggest a potentially underrecognised role for *BCOR* and *NCOR2* alterations in the epigenetic dysregulation of cutaneous CD30+ T LPD.

The most frequently altered gene in our cohort was not an epigenetic modifier, but *KDR*, a receptor for VEGF, occurring in 24% of cases. Upon activation, KDR promotes downstream MAPK and PI3K signalling, driving cell cycle progression.^51^ To our knowledge, *KDR* alterations have not previously been described in association with CD30+ T LPD. The most common variant identified, p.Gln472His, has also been associated with improved progression-free and overall survival in a large cohort of patients with diffuse large B cell lymphoma.^52^ In our study, *KDR* variants showed a trend toward enrichment in the low-risk group, although this did not reach statistical significance, likely due to the limited number of cases harbouring these alterations. Despite this, GSEA demonstrated that *KDR*-mutated cases were associated with increased G protein-coupled receptor signalling, supporting a functional impact of these variants. Consistent with this observation, low-risk cases were also enriched for G protein-coupled receptor signalling in analyses comparing risk groups, further suggesting a potential link between *KDR* alterations and a less aggressive disease phenotype.

Mutations in intracellular signalling pathways, including JAK/STAT, PI3K/AKT, and G protein signalling, have been reported in CD30+ T LPDs.^46^, ^53^ GSEA and Enrichr confirm their activity in this cohort, with JAK/STAT enriched in the high-risk group and PI3K/AKT in the low-risk group. In contrast to prior studies, we observed relatively few mutations in canonical components of the JAK/STAT pathway. This discrepancy is likely attributable to the heterogeneity of cases included in our study, which may dilute subsets enriched for such alterations. For example, even in large genomic studies of CD30+ nodal T-cell lymphomas, JAK/STAT mutations are confined to a specific subset.^54^ Similarly, studies reporting recurrent JAK/STAT alterations have examined more narrowly defined cohorts, such as cases co-expressing CD30 and CD15.^55^ Another study focusing on JAK2 fusions in cutaneous T LPDs included predominantly less aggressive cases with limited need for systemic therapy, reflecting a biologically distinct population from that included in our study.^56^ In addition, certain well-defined subtypes, such as primary cutaneous CD8-positive aggressive epidermotropic cytotoxic T-cell lymphoma, known to be enriched for *JAK2* fusions, are not included in this study.^57^ Taken together, the clinical and biological heterogeneity of our cohort likely accounts for the lower observed frequency of JAK/STAT pathway mutations.

Gene fusions were identified in a minority of cases (4/44), but, notably, they all occurred in the high-risk group, suggesting a possible association with more aggressive disease. Both *NPM1::ALK* and *NPM1::TYK2* are recognised recurrent fusions in nALCL and cALCL, respectively.^58^ *PRMT1::TYK2* is a novel fusion found in a case of transformed MF, which may similarly promote oncogenesis through constitutive TYK2 activation. In addition, a *PARG::BMS1* fusion was detected, which has been previously reported in ALCL^59^ and other tumour types,^60^, ^61^ here seen in a case of cALCL also in the high-risk group.

Mutational signature profiles were shared across risk groups, but provided insight into disease pathogenesis. In our cohort, where all cases have cutaneous involvement, 34% of cases exhibited a UV-associated signature, accounting for 11.9% of the total mutational burden. This percentage is within the wide range of estimates seen in CTCL, where the etiologic role of UV exposure remains debated.^62^, ^63^ Notably, the ALCL case of uncertain primary origin (cutaneous vs. nodal) showed a strong UV signature, raising the possibility of a skin-origin clone with subsequent nodal spread, although this cannot be established definitively.

Across risk groups, there were recurrent age-related (5-methylcytosine deamination), DNA mismatch repair deficiency (dMMR), and POLE-associated signatures. POLE and dMMR signatures are linked to high mutational burden and increased neoantigen load and predict response to immune checkpoint inhibition in several solid tumours including colorectal and endometrial cancers.^64^, ^65^ By analogy, these signatures may have therapeutic relevance in CD30+ T LPD, a possibility that warrants further clinical investigation.

Although gene mutations and mutational signatures did not differ between high- and low-risk disease, the groups diverged in gene expression profiles and immune microenvironment.

Using bulk transcriptomic data to perform immune cell deconvolution, the low-risk group of CD30+ T LPD was found to be associated with a greater abundance of resting dendritic cells compared to the high-risk group. While dendritic cells have a critical role in antigen presentation and immune surveillance, resting dendritic cells are in an immature or inactive state, and have reduced ability to produce pro-inflammatory cytokines. Such dendritic cells have been shown to promote immune tolerance in the lymphoma microenvironment.^66^ Their abundance in the low-risk clinical group is notable, and may be reflective of the less inflammatory nature of the skin lesions in these patients.

Gene expression analysis showed elevated expression of genes associated with cell cycle progression, DNA damage response, and immune activation in the high-risk group compared to the low-risk group. GSEA showed upregulation of JAK/STAT target genes (canonically associated with cytokine and interferon signalling), MYC targets, and the G2M checkpoint gene set. Together, these results indicate increased cytokine-driven signalling and increased oncogenic cell cycle progression in the high-risk patients. *MYC*'s role as a proto-oncogene is well known, including in aggressive B cell lymphomas. Its expression has also been documented in a variety of T cell malignancies.^67^ In MF, *MYC* expression is increased in advanced stage disease compared with early stage disease.^68^ While the above studies employed immunohistochemistry to determine MYC expression, in our study of CD30+ T LPD, GSEA further elucidates the role of *MYC* on downstream gene targets in high-risk disease compared to low-risk disease.

Gene expression analysis in this study also adds to prior work showing that derangement of cell cycle checkpoints is likely part of the pathogenesis in CTCL, and may confer more aggressive disease.^35^, ^69^ Aurora kinase A, a serine/threonine kinase that regulates the G2/M transition, was previously reported to be upregulated in CTCL^35^ and was also upregulated in our cohort of adverse risk CD30+ T LPD, suggesting that it may be a good therapeutic target in cutaneous CD30+ T LPD.

Systematic analysis of gene overexpression in the high-risk group compared to the low-risk group allows for the identification of other potential therapies for the high-risk patients who are in urgent need of alternative treatment options. Of the genes that are statistically significantly overexpressed in the high-risk group, six (*BIRC3, PDE4D, IL2RA, POLR2B, CUL1,* and *PRKDC*) represent potential therapeutic targets for drugs that are either commercially available or are in clinical development. Most notably, our data identified *IL2RA,* which is targeted by denileukin diftitox, an FDA approved therapy for relapsed refractory CTCL. It carries a black box warning of potentially fatal capillary leak syndrome, making it desirable to identify alternative therapies for these patients. Based on these gene expression data, several other drugs may be considered for study in cutaneous CD30+ T LPD. Roflumilast is a PDE4 inhibitor approved for chronic obstructive pulmonary disease and is used in topical form for the treatment of plaque psoriasis, but has also been shown to have some efficacy in B cell malignancies.^70^ Lurbinectedin, FDA approved for metastatic small cell lung cancer, targets RNA polymerase II for degradation and inhibits oncogenic transcription.^71^ *BIRC3* may be targeted using SMAC mimetics such as birinapant, a drug in clinical development for solid malignancies such as head and neck cancer.^72^ *CUL1* may be indirectly targeted using pevonedistat, a drug studied in a phase 3 trial in myeloid malignancies that inhibits the activation of cullin 1 through the neddylation pathway.^73^ Finally, for *PRKDC* overexpression, peposertib is a drug in clinical development for various solid malignancies that inhibits DNA-activated protein kinase.^74^ These expression data offer a mechanistic rationale for evaluating these therapeutic strategies.

Cutaneous CD30+ T LPD comprise a heterogeneous group of entities with variable clinical outcomes. In this study, 79 patients were stratified into two clinical risk groups based on treatment recommendations, and lesional skin biopsies were analysed for mutational drivers, gene fusions, transcriptional profiles, mutational signatures, and the tumour immune microenvironment. Although using treatment recommendations as a proxy for disease aggressiveness has inherent limitations given potential confounding by patient age, comorbidities, and physician recommendation, the observed survival difference between groups remained robust despite this potential bias toward the null. The retrospective design and reliance on archival tissue further limited sample availability, with many specimens insufficient for comprehensive molecular profiling, resulting in incomplete datasets and precluding the ability to form a validation cohort.

Despite these limitations, this series of patients with cutaneous involvement by CD30+ T LPD provides an extensive and in-depth genomic characterisation of the disease spectrum with detailed clinical correlation. This dataset highlights the recurrent genetic drivers and mutational signatures in CD30+ T LPD and shows the transcriptional and immunological differences between high-risk and low-risk cohorts. Future longitudinal studies are needed to validate the prognostic findings. The CD30+ T LPD are a highly heterogeneous group of diseases that should be better molecularly characterised in clinical practice, and novel rational combination therapies targeting specific cellular mechanisms (e.g. epigenetics, intracellular signalling, cell cycle progression, and immune checkpoints) need to be evaluated prospectively through clinical trials.

## Contributors

All authors have read and approved the final version of the manuscript. Dennis Owusu and Devang Thakkar have accessed and verified the underlying data.

Conceptualisation: Magdalena Czader, Kikkeri N. Naresh, Sandeep S. Dave, and Jie Wang. Methodology: Leonardo P.A. Biral, Veronica Russell, Cassandra Love, Dennis Owusu, Elizabeth Thacker, Devang Thakkar, Lanie Happ, and Jie Wang. Investigation: Leonardo P.A. Biral, Veronica Russell, Maria Angelica Selim, Caroline Roth, Cassandra Love, Jean L. Koff, Chee Leong Cheng, Sirpa Leppä, Kikkeri N. Naresh, and Jie Wang. Validation: Dennis Owusu, Devang Thakkar. Formal Analysis: Leonardo P.A. Biral, Veronica Russell. Software: Clay Parker. Resources: Maria Angelica Selim, Emily Mason, Alexandra Kovach, Catalina Amador, Jean L. Koff, David Jaye, Chee Leong Cheng, Andrew Evans, Andrew Evens, Raju Pillai, Sirpa Leppä, Eric D. Hsi, Sarah Ondrejka, Matthew McKinney, Chad M. McCall, Shelby Bettoney, Payal Sojitra, Amir Behdad, Carlos Galvez, Yuri Fedoriw, Magdalena Czader, Rohit Gulati, Kikkeri N. Naresh, and Jie Wang. Data Curation: Leonardo P.A. Biral, Veronica Russell, Caroline Roth, Cassandra Love, Ayush Batra, Emily Mason, Alexandra Kovach, Catalina Amador, Jean L. Koff, David Jaye, Chee Leong Cheng, Andrew Evans, Andrew Evens, Raju Pillai, Sirpa Leppä, Eric D. Hsi, Sarah Ondrejka, Matthew McKinney, Chad M. McCall, Shelby Bettoney, Payal Sojitra, Amir Behdad, Carlos Galvez, Yuri Fedoriw, Clay Parker, Magdalena Czader, Rohit Gulati, Kikkeri N. Naresh, and Jie Wang. Writing—Original Draft: Leonardo P.A. Biral, Jie Wang. Writing—Review & Editing: Leonardo P.A. Biral, Maria Angelica Selim, Jennifer Shingleton, Jean L. Koff, Sirpa Leppä, Kikkeri N. Naresh, Sandeep S. Dave, and Jie Wang. Project Administration: Caroline Roth, Jennifer Shingleton, Kikkeri N. Naresh, and Sandeep S. Dave. Funding Acquisition: Sandeep S. Dave, Jie Wang. Supervision: Sandeep S. Dave.

## Data sharing statement

De-identified DNA and RNA sequencing data will be publicly available through the European Genome Archive (EGA, ega-archive.org) upon publication (no end date). Sequencing data are deposited under the following ID numbers: DNA sequencing, EGAD50000001982; RNA sequencing, EGAD50000001983.

## Declaration of interests

D.T., C.L., J.S., E.T., C.P., L.H., and S.S.D. own equity in Data Driven Bioscience.

D.T., E.T., C.P. and L.H. are employed by Data Driven Bioscience.

C.L., J.S., and L.P.A.B. receive compensation as consultants from Data Driven Bioscience.

C.M.M. has received compensation as a consultant from Scopio, Ltd, serves as the president-elect of the North Carolina Society of Pathologists (unpaid role) and serves as the Vice-Chair (Hematology and Clinical Microscopy Committee) of the College of American Pathologists (unpaid role).

A.Eve. has received honoraria for consulting services from Novotech, Novartis, Pharmacyclics, Pfizer, Genentech and CRISPR.

M.C. has received honoraria and travel support from Roche and has served on a committee for the Society for Hematopathology (unpaid).

A.B. has received compensation from Lilly for consulting fees and honoraria for presentations.

J.W. has received honoraria for serving on an Advisory Board for Acrotech Biopharma.

Other authors declare no competing financial interests.

## Appendix A Supplementary data

Supplementary Figures **Supplemental Figure S1: Diagram of samples used in each analysis.** Out of 79 patient samples accrued, 74 had accompanying survival data. 53 out of 79 samples had sufficient material to undergo sequencing. Of these, 44 samples passed RNAseq quality control metrics and were included in gene expression analyses. 38 out of 53 samples passed DNAseq quality control metrics and were included in mutation analyses. **Supplemental Figure S2: (A)** Overall survival Kaplan-Meier curve stratified by clinical risk group, excluding LyP and nALCL cases. Number of patients at risk and 95% CIs shown. We observe significant differences in survival between the low (green) and high (orange) risk groups (p=0.001 [log-rank]). **(B)** Confusion matrix showing concordance of assigning patient risk group by treatment (Low-Risk vs. High-Risk) compared to the presence or absence of tumour or diffuse skin involvement. The two methods were concordant for 36/54 patients. **(C)** Overall survival is not significantly different between groups with and without tumour or diffuse skin involvement. (p=0.12 [two-tailed log-rank]) Number of patients at risk and 95% CIs shown. **Supplemental Figure S3: Lolliplots of variants in genes involved in epigenetic regulation. (A)***BCOR*, **(B)***NCOR2*, **(C)***PRDM1*, **(D)***TET2*. Mutation types are indicated by coloured circles (green, frameshift indel; red, nonsynonymous SNV; blue, stopgain). Amino acid changes are indicated above each circle. The number of each mutation identified across the cohort is indicated by the height of each bar. For lolliplots with no y-axis, the height of each bar is 1 mutation. X-axis indicates amino acid positions. Protein domains are indicated by coloured rectangles and labeled below each plot. Indel, short insertion/deletion. SNV, single nucleotide variant. **Supplemental Figure S4: Lolliplots of variants in tyrosine kinases and proliferation genes. (A)***EPHB1*, **(B)***GNAS*, **(C)***JAK3*, **(D)***TGFBR2*. Mutation types are indicated by coloured circles (yellow, nonframeshift indel; red, nonsynonymous SNV). Amino acid changes are indicated above each circle. The number of each mutation identified across the cohort is indicated by the height of each bar. For lolliplots with no y-axis, the height of each bar is 1 mutation. X-axis indicates amino acid positions. Protein domains are indicated by coloured rectangles and labeled below each plot. Indel, short insertion/deletion. SNV, single nucleotide variant. **Supplemental Figure S5: Lolliplots of variants in cytokine signalling suppressor genes. (A)***SH2B3*, **(B)***SOCS1*. Mutation types are indicated by coloured circles (yellow, nonframeshift indel; red, nonsynonymous SNV). Amino acid changes are indicated above each circle. Each mutation was identified once in the cohort. X-axis indicates amino acid positions. Protein domains are indicated by coloured rectangles and labeled below each plot. Indel, short insertion/deletion. SNV, single nucleotide variant. **Supplemental Figure S6: Lolliplots of variants in tumour suppressor genes. (A)***DUSP22*, **(B)***PTEN*, **(C)***WT1*. All mutations are nonsynonymous single nucleotide variants (SNV). Amino acid changes are indicated above each circle. Each mutation was identified once in the cohort. X-axis indicates amino acid positions. Protein domains are indicated by coloured rectangles and labeled below each plot. **Supplemental Figure S7: Lolliplots of variants in DNA damage response genes. (A)***ATM*, **(B)***MSH6*. All mutations are nonsynonymous single nucleotide variants (SNV). Amino acid changes are indicated above each circle. Each mutation was identified once in the cohort. X-axis indicates amino acid positions. Protein domains are indicated by coloured rectangles and labeled below each plot. **Supplemental Figure S8: Lolliplots of variants in immune evasion genes. (A)***CD58*, **(B)***CIITA*. Mutation types are indicated by coloured circles (red, nonsynonymous SNV; black, startloss). Amino acid changes are indicated above each circle. Each mutation was identified once in the cohort. X-axis indicates amino acid positions. Protein domains are indicated by coloured rectangles and labeled below each plot. SNV, single nucleotide variant.
