# Supplementary material for: Characterising the genomic landscape of two clinical risk groups in CD30+ T-cell lymphoproliferative disorders with cutaneous involvement: a multi-institutional retrospective study
Source: eBioMedicine. 2026 Oct 27;133:106532. doi: 10.1016/j.ebiom.2026.106532 (PMC13733320; doi:10.1016/j.ebiom.2026.106532)

Supplementary Materials for "Characterizing the Genomic Landscape of Two Clinical Risk Groups in CD30+ T-Cell Lymphoproliferative Disorders with Cutaneous Involvement: A Multi-Institutional Retrospective Study"

Contents:

Supplemental Figure S1: Diagram of samples used in each analysis 2

Supplemental Figure S2 3

Supplemental Figure S3: Lolliplots of variants in genes involved in epigenetic regulation 4

Supplemental Figure S4: Lolliplots of variants in tyrosine kinases and proliferation genes 5

Supplemental Figure S5: Lolliplots of variants in cytokine signalling suppressor genes 6

Supplemental Figure S6: Lolliplots of variants in tumour suppressor genes 7

Supplemental Figure S7: Lolliplots of variants in DNA damage response genes 8

Supplemental Figure S8: Lolliplots of variants in immune evasion genes 9

Supplemental Figure S1

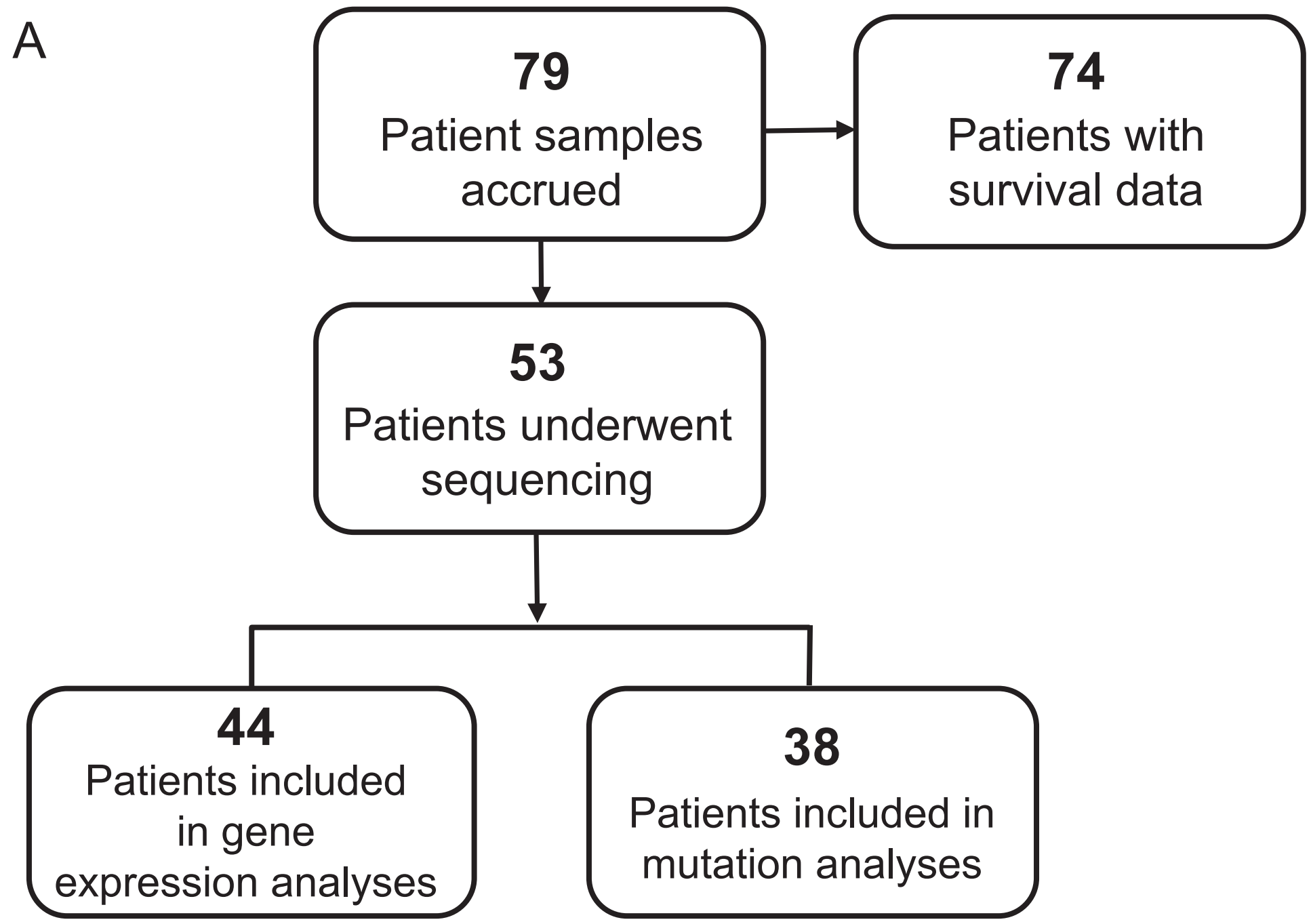

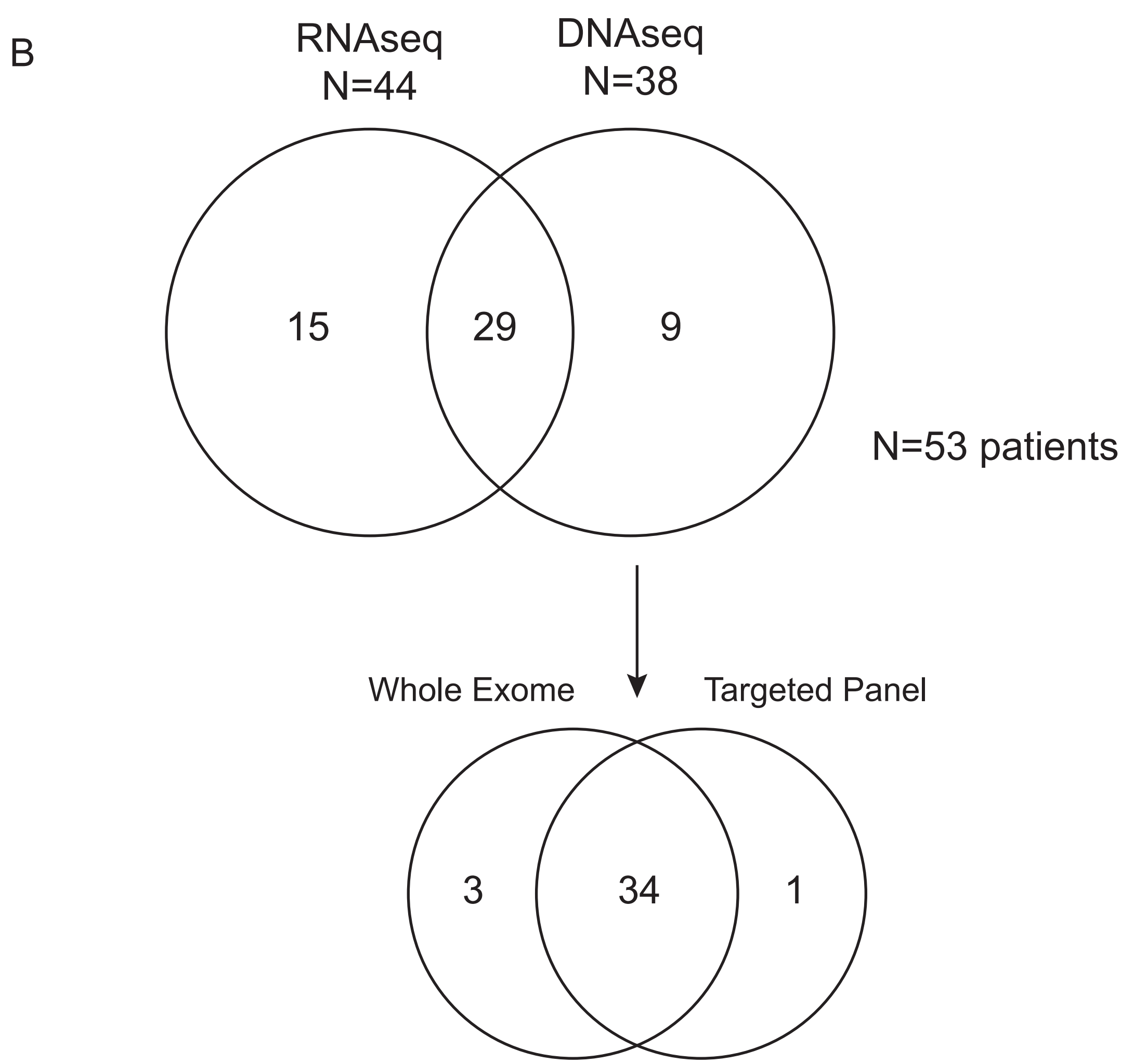

Supplemental Figure S2

A

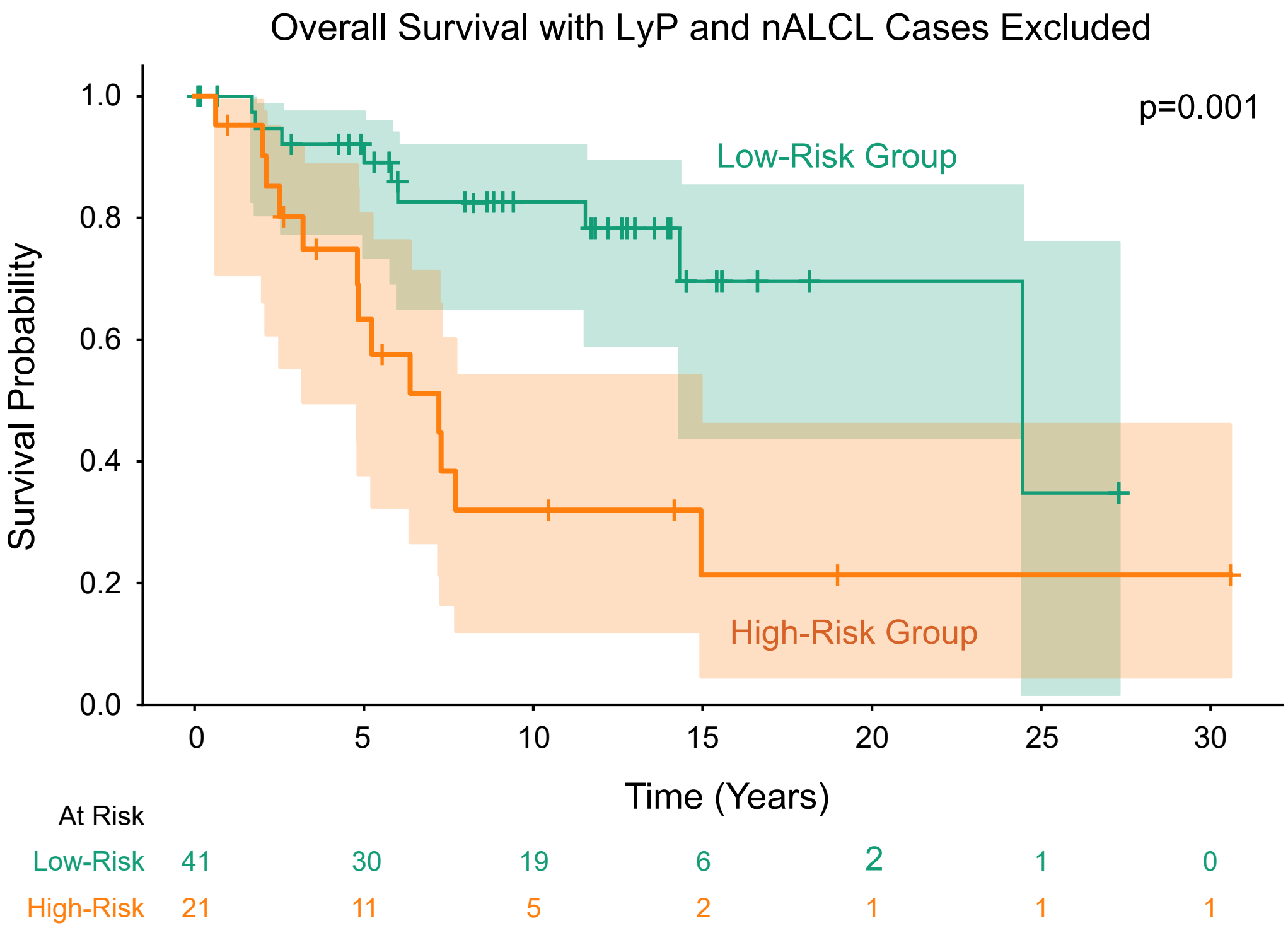

B

	Low-Risk	High-Risk
No tumour or diffuse skin involvement	17	4
Tumour or diffuse skin involvement	14	19

C

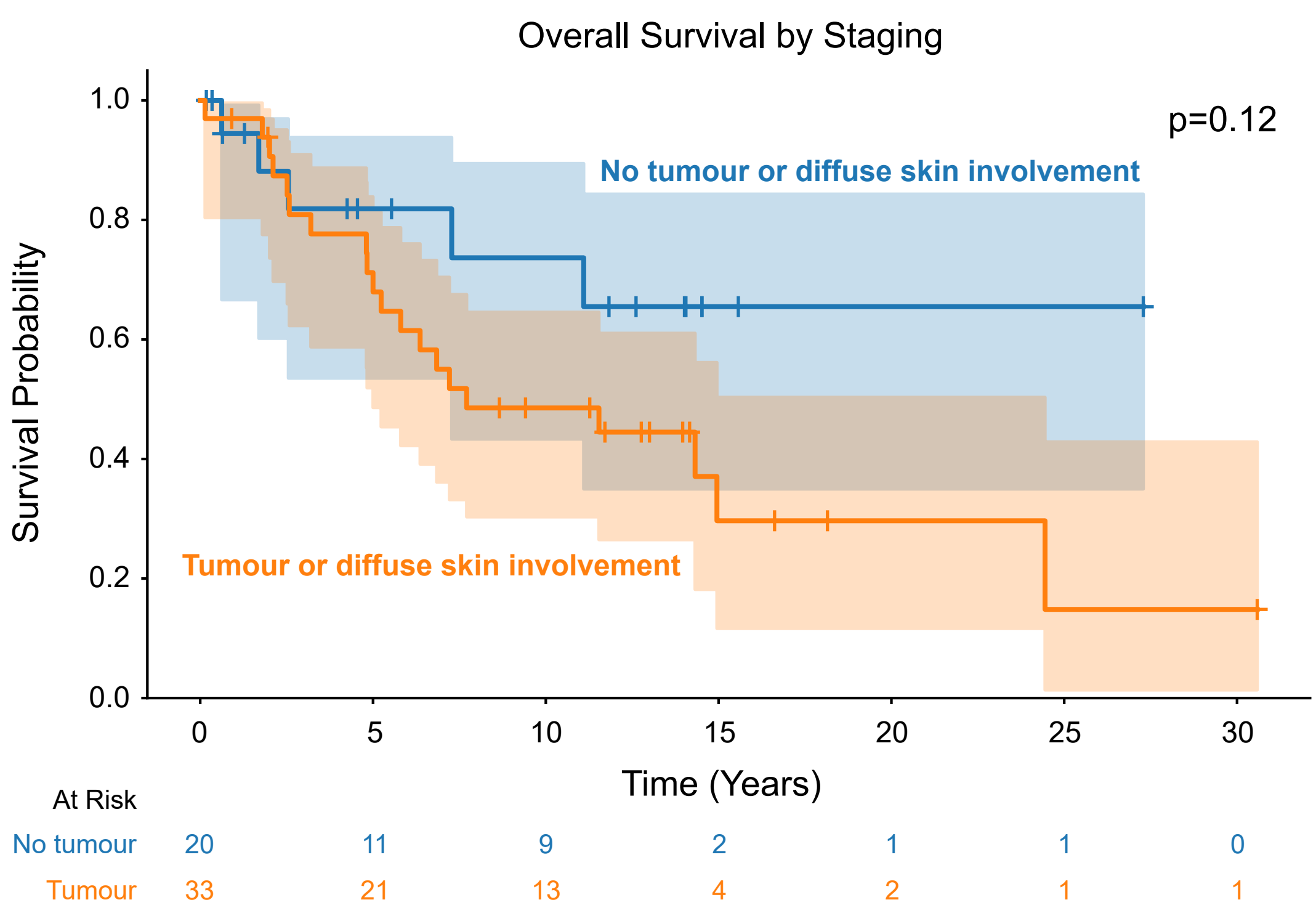

Supplemental Figure S3

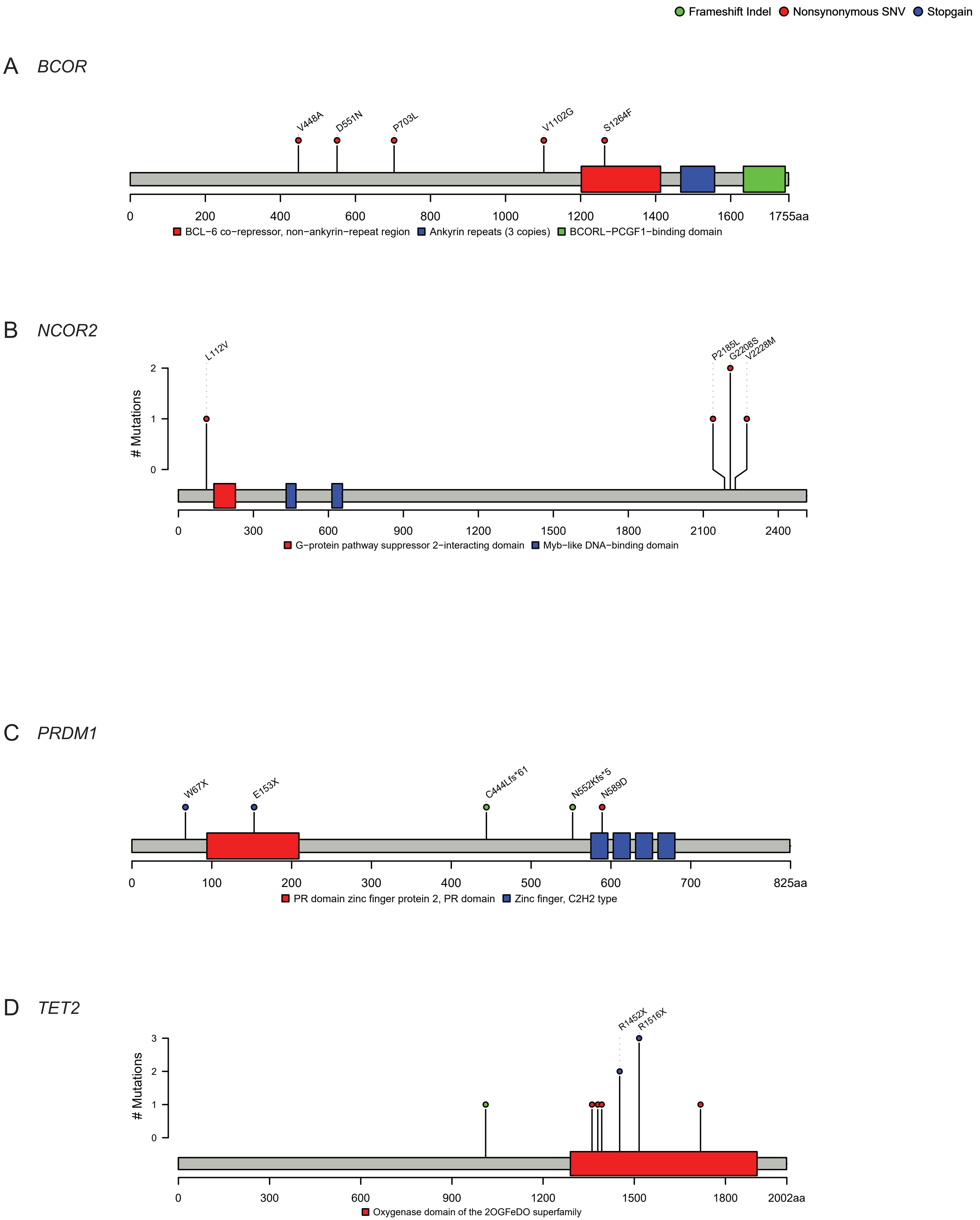

Supplemental Figure S4

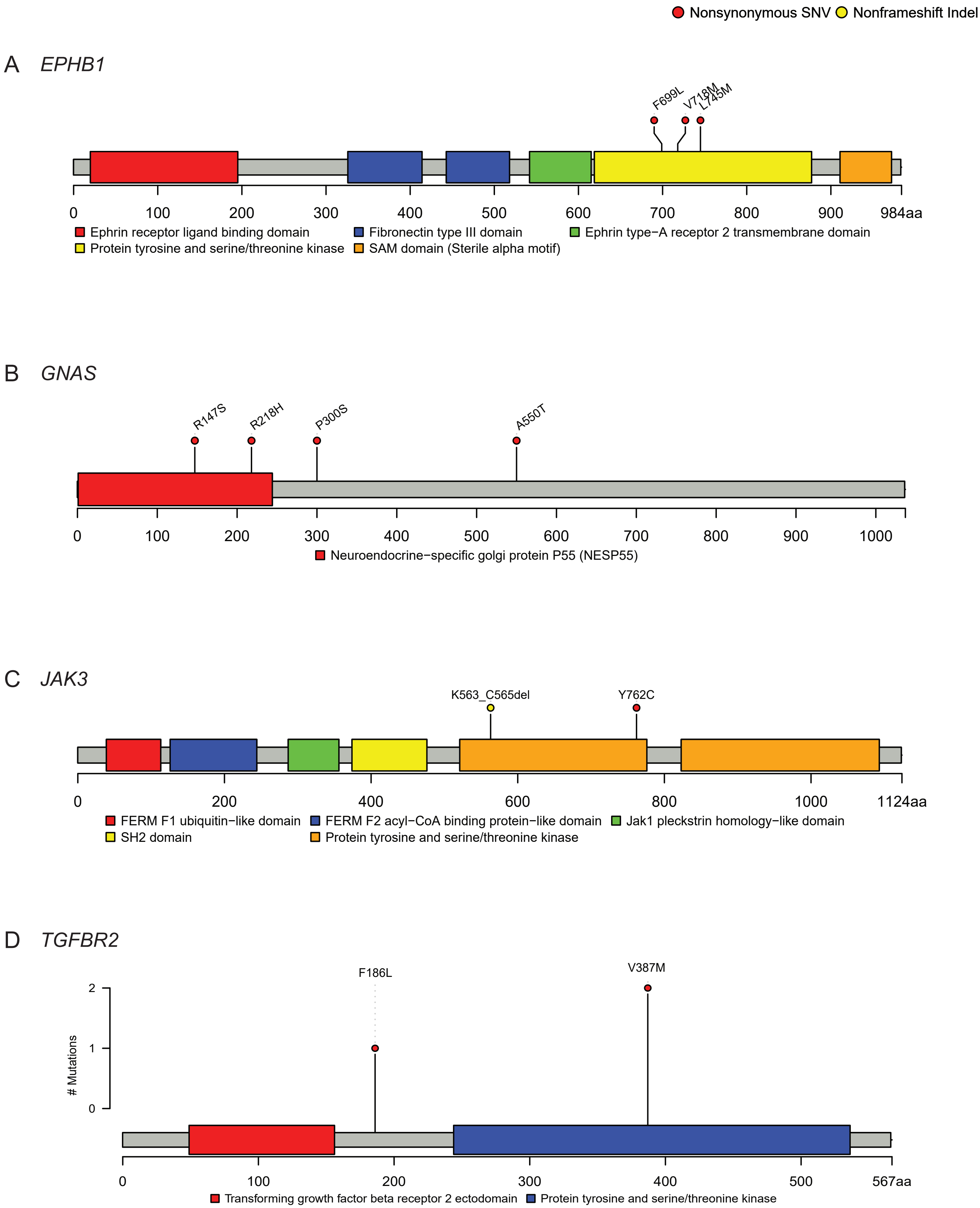

Supplemental Figure S5

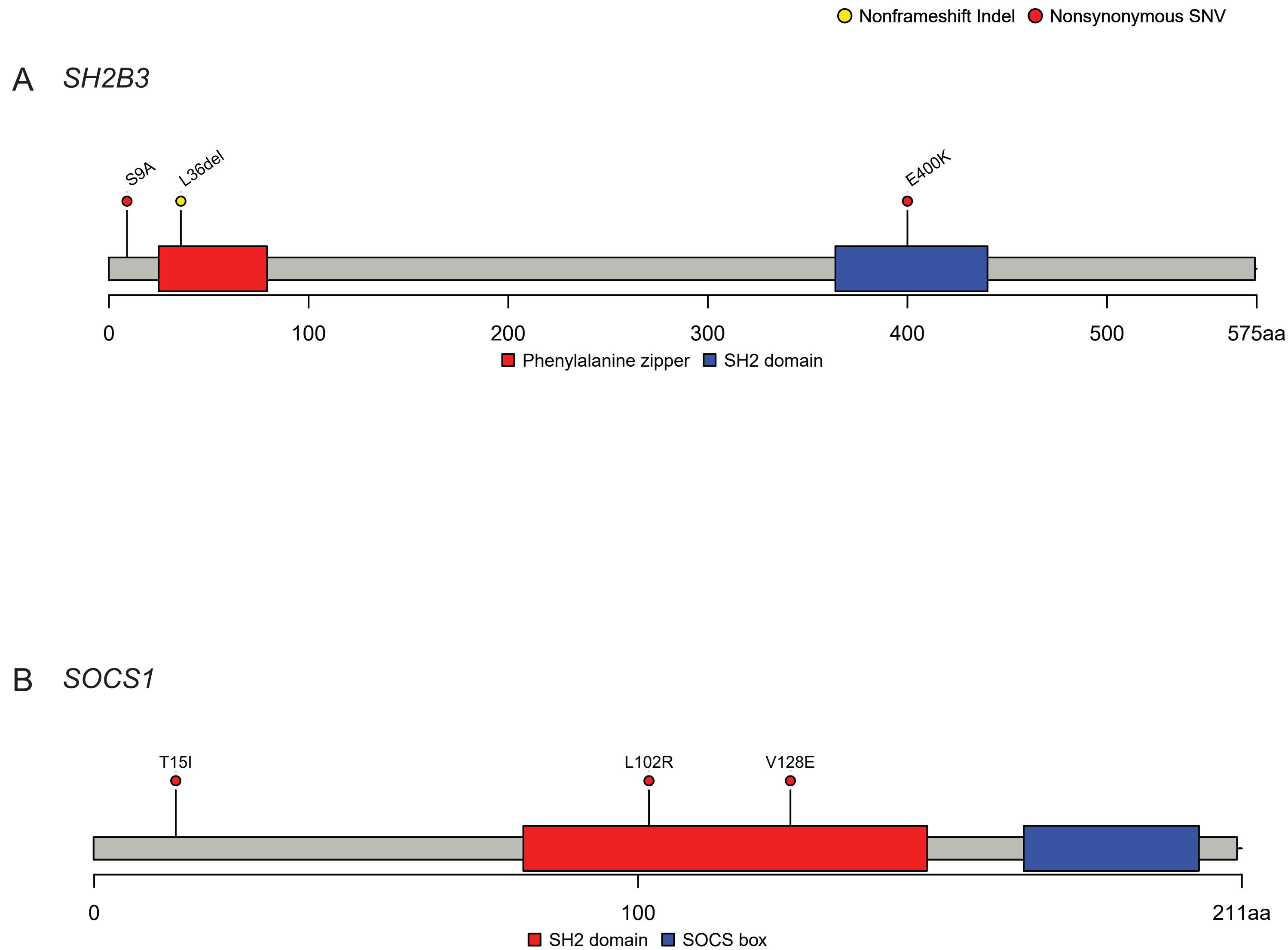

Supplemental Figure S6

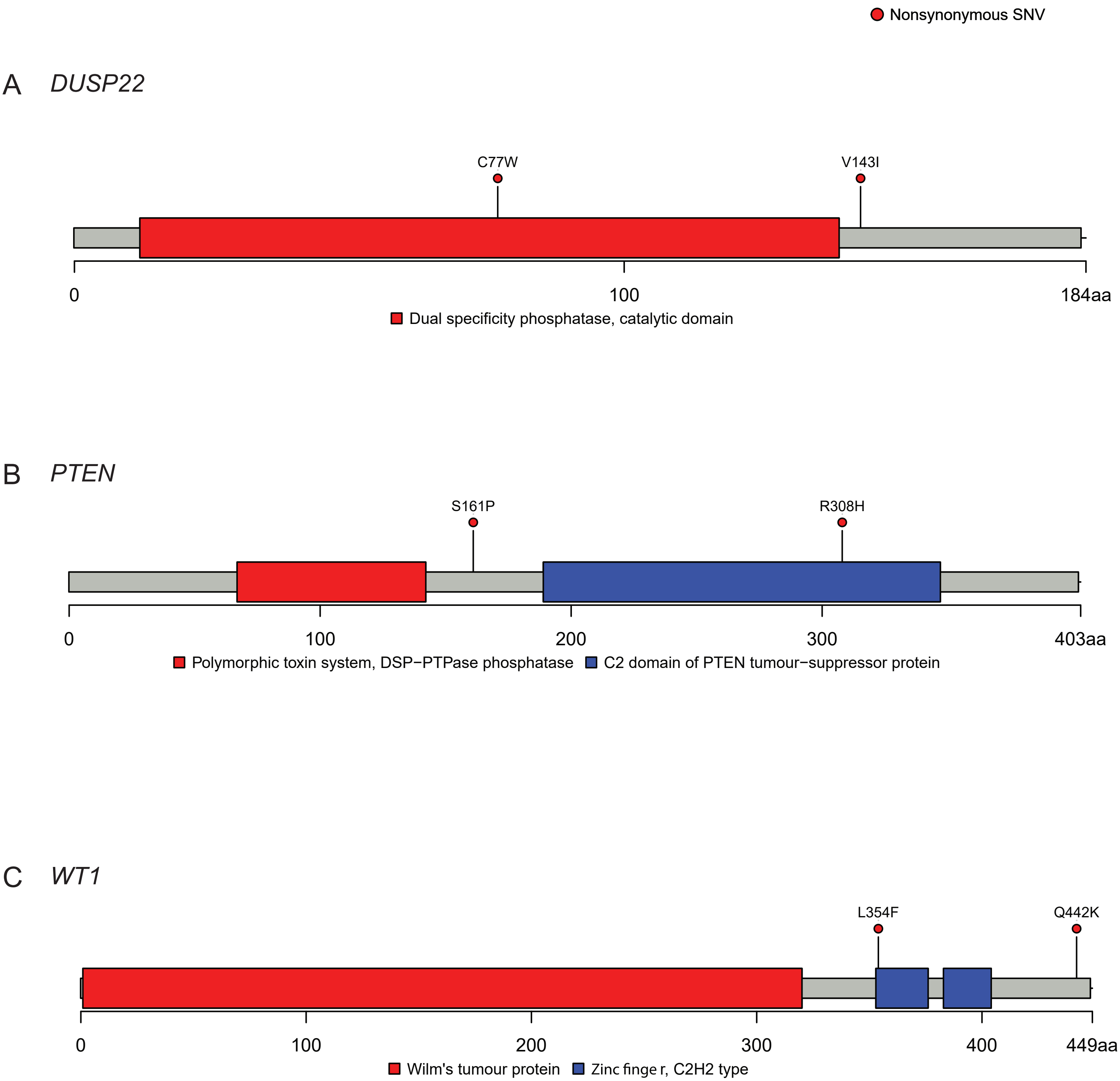

Supplemental Figure S7

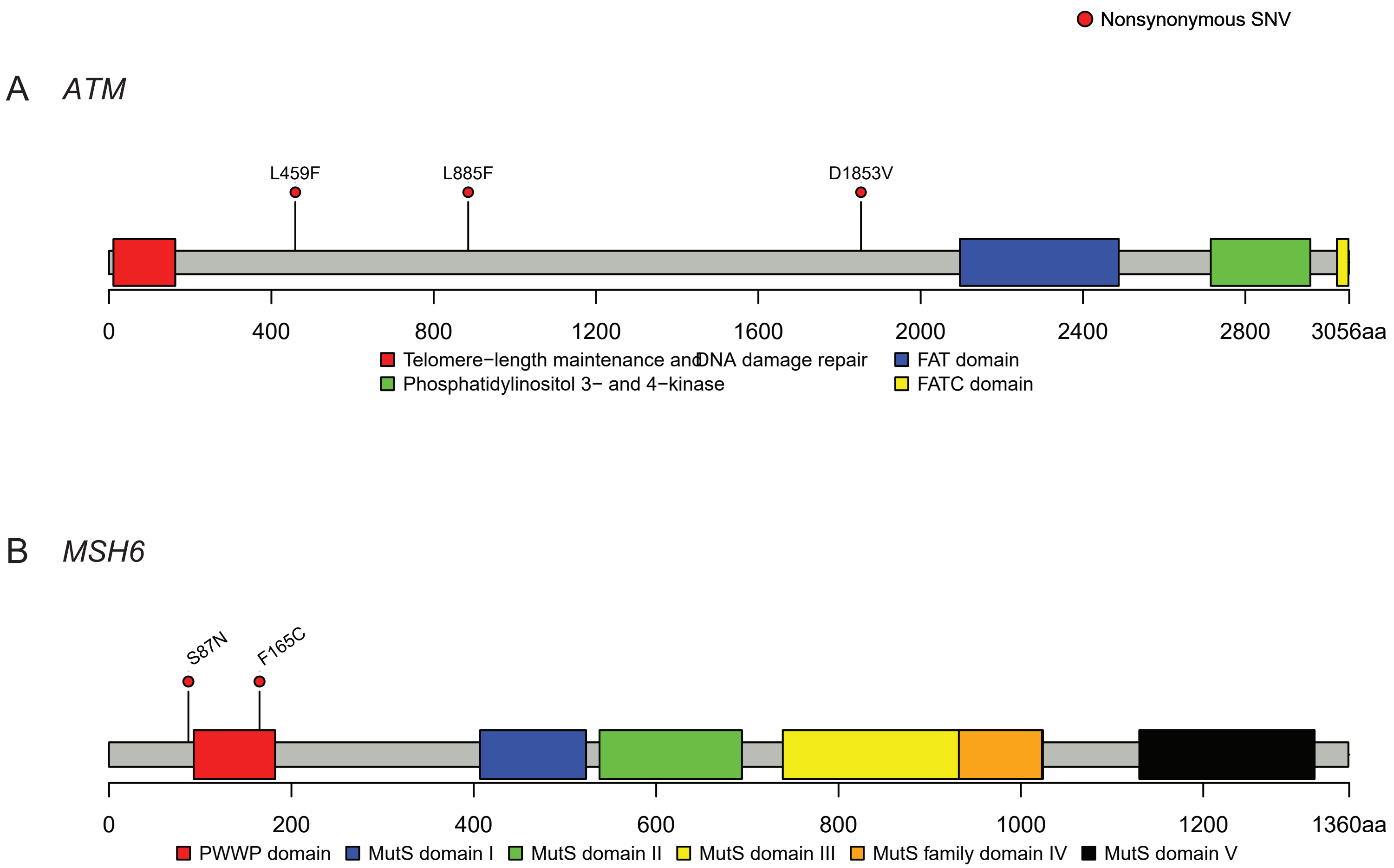

Supplemental Figure S8

A *CD58*

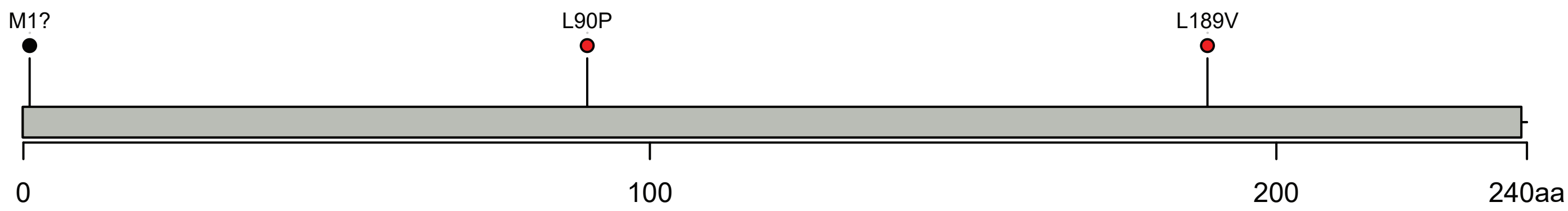

B *CIITA*

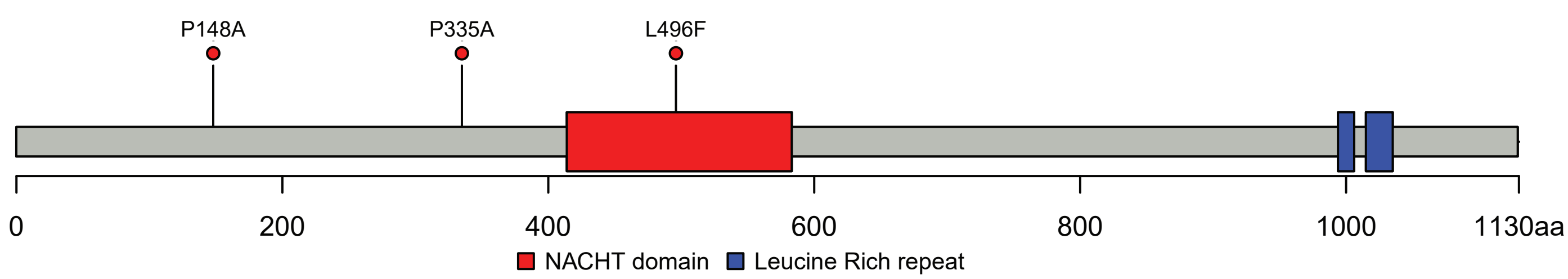

Supplement: Supplementary file 1 [file mmc1.pdf]
